# Two NCA1 isoforms interact with catalase in a mutually exclusive manner to redundantly regulate its activity in rice

**DOI:** 10.1186/s12870-019-1707-0

**Published:** 2019-03-18

**Authors:** Jianzhe Liu, Lili Cui, Zongwang Xie, Zhisheng Zhang, Ee Liu, Xinxiang Peng

**Affiliations:** State Key Laboratory for Conservation and Utilization of Subtropical Agro-bioresources, College of Life Sciences, South China Agricultural University, Guangzhou, 510642 Guangdong China

**Keywords:** Catalase, Function, Interaction, Isoforms, NCA1, Rice

## Abstract

**Background:**

NCA1 (NO CATALASE ACTIVITY 1) was recently identified in *Arabidopsis* as a chaperone protein to regulate catalase (CAT) activity through maintaining the folding of CAT. The gene exists mainly in higher plants; some plants, such as *Arabidopsis*, contain only one *NCA1* gene, whereas some others such as rice harbor two copies. It is not yet understood whether and how both isoforms have functioned to regulate CAT activity in those two-copy-containing plant species.

**Results:**

In this study, we first noticed that the spatiotemporal expression patterns of *NCA1a* and *NCA1b* were very similar in rice plants. Subsequent BiFC and yeast three-hybrid experiments demonstrated that both NCA1a and NCA1b show mutually exclusive, rather than simultaneous, interaction with CAT. For a further functional analysis, *nca1a* and *nca1b* single mutants or double mutants of rice were generated by CRISPR/Cas9. Analysis on these mutants under both normal and salinity stress conditions found that, as compared with WT, either *nca1a* or *nca1b* single mutant showed no difference at phenotypes and CAT activities, whereas the double mutants constantly displayed very low CAT activity (about 5%) and serious lesion phenotypes.

**Conclusions:**

These results suggest that NCA1a and NCA1b show mutually exclusive interaction with CAT to regulate CAT activity in a functionally-redundant manner in rice.

**Electronic supplementary material:**

The online version of this article (10.1186/s12870-019-1707-0) contains supplementary material, which is available to authorized users.

## Background

Catalase (CAT) is a heme-containing enzyme which typically catalyzes the dismutation of H_2_O_2_ to produce H_2_O and O_2_ in plants [1]. CAT usually consists of polypeptides of 50–70 kDa in mass that are organized into tetramers, with each monomer bearing a heme prosthetic group [2]. Known to date, all plants, including monocots and dicots such as tobacco, *Arabidopsis*, maize, pumpkin, and rice, contain three CAT genes [1]. The three genes of *Arabidopsis*, named as *CAT1,* C*AT2*, and *CAT3*, encode three corresponding proteins which consist of 492 amino acids, with high similarity between the amino acid sequences. The three genes, identified in the rice genome, were named as *CATA, CATB* and *CATC*, respectively [3]. Comparisons of gene structures and functions found that OsCATA corresponds to AtCAT3, OsCATB to AtCAT1, and OsCATC to AtCAT2 [4].

*AtCAT1* is mainly expressed in pollen and seeds, while *AtCAT2* is predominantly expressed in photosynthetic active tissues and *AtCAT3* in vascular tissues and senescent leaves [4]. CAT is known to exist in the peroxisome although evidence emerged that CAT activity was detected in different organelles [4]. While *AtCAT3* knockout only slightly reduced CAT activity; deletion of *AtCAT2* reduced the activity by 80%; on the contrary while deletion of *AtCAT1* had no effect on CAT activity [4, 5]. No obvious phenotypes were observed in the *CAT1* or *CAT3-*deficeint *Arabidopsis*, whereas the *cat2* mutant displayed defects in many processes, including photorespiration and pathogenesis, salicylic acid-dependent hypersensitive response-like lesion formation [6], autophagy-dependent cell death [7], and altered gene expressions during both biotic and abiotic stresses, including cold, heat, and drought [5, 8]. In rice, the role of the three isoforms in catalase activity is poorly understood. Our recent results showed that CATC accounts for majority of CAT activities in rice leaves [9], and its knockout mutant exhibited cell-death phenotype, similar to the *cat2* mutant of *Arabidopsis* [10].

Plant CAT is a key antioxidant enzyme indispensable for plants to cope with adverse environmental stresses, thus, understanding how catalases are regulated is particularly important. Regulation at its transcriptional levels has been extensively investigated and many factors are shown to be involved in the regulation, such as circadian rhythm, reactive oxygen species (ROS), senescence and ABA signaling [11–14]. CAT can be also regulated at the protein level. In *Arabidopsis*, LSD1 (LESION SIMULATING DISEASE 1), a zinc finger protein, affected programmed cell death through interacting with CAT proteins in the cytosol and altering their activities [15, 16]. Hsp17.6CII, a peroxisomal small heat shock protein and chaperone, was shown to interact with CAT2 in peroxisome and to increase CAT activity [17]. In addition, CAT3 activity may be modulated through protein phosphorylation by CPK8 in response to drought stress [18]. Calmodulin [19], nucleoside diphosphate kinase 1 [20], salt overly sensitive 2 (SOS2) [21] and triple gene block protein 1 [22] have been also reported to be able to interact with CAT. But how these interaction proteins regulate CAT is still not well known. NCA1 (NO CATALASE ACTIVITY 1) was recently identified in *Arabidopsis*, which regulates CAT2 activity in response to salt, cold, and high pH. NCA1 acts as a chaperone protein that may fold catalase to a functional structure, the binding of a zinc ion in the N-terminal RING-finger domain of NCA1 is essential for the full function of CAT2 and that the tetratricopeptide repeat (TPR) domain in the NCA1 C terminus mediates interaction with CAT2 [5, 7]. More interestingly, NCA1 interacts with CAT in the cytosol, whereas the CAT function is carried out in the peroxisome [5]. Some plant species contain only one NCA1 gene, while some have two homologs in the genome. Whether the two isoforms in those two-copy-containing plant species function equally or differently in regulating CAT activity and how they realize the function remains to be investigated.

As a monocotyledonous model plant, rice harbors two NCA1 genes, so it is particularly interesting to address the above question in rice. In this study, the rice two NCA1 isoforms were comparatively investigated in terms of function and mechanism. The results demonstrated that NCA1a and NCA1b show mutually exclusive interaction with CAT to regulate its activity in a functionally-redundant manner in rice.

## Results

### *NCA1* genes exist mainly in higher plants

*NCA1* was first identified in *Arabidopsis*, with only one gene in its genome [7], whereas two *NCA1* genes are identified in the rice genome, hereby named as Os*NCA1a* (Os01g0104100) and Os*NCA1b* (Os02g0795300), both the mRNA sequences and polypeptides of these three *NCA1* genes are highly similar (Additional file 1 Table S1). To examine the evolution of OsNCA1a and OsNCA1b, a phylogenetic tree was generated using the full-length protein sequences of OsNCA1a and OsNCA1b as input. It was found that some species have one NCA1 gene and some have two. Most of the organisms with the NCA1 gene are higher plants (Additional file 2 Figure S1). No NCA1 homologous gene was found in *Homo sapiens*, *Musmusculus*, *Drosophila melanogaster*, *Caenorhabditis*, *Saccharomyces cerevisiae, Escherichia coli*, *Zosteramarina*, *Spirodelapolyrhiza*, *Sphagnum*, *Ostreococcuslucimarinus*, *Oropetium*, *Micromonaspusilla*, *Marchantiapaleacea*, *Dunaliella Salina*, *Coccomyxasubellipsoidea*.

### Spatiotemporal expression patterns and subcellular localization

**Fig. 1:**
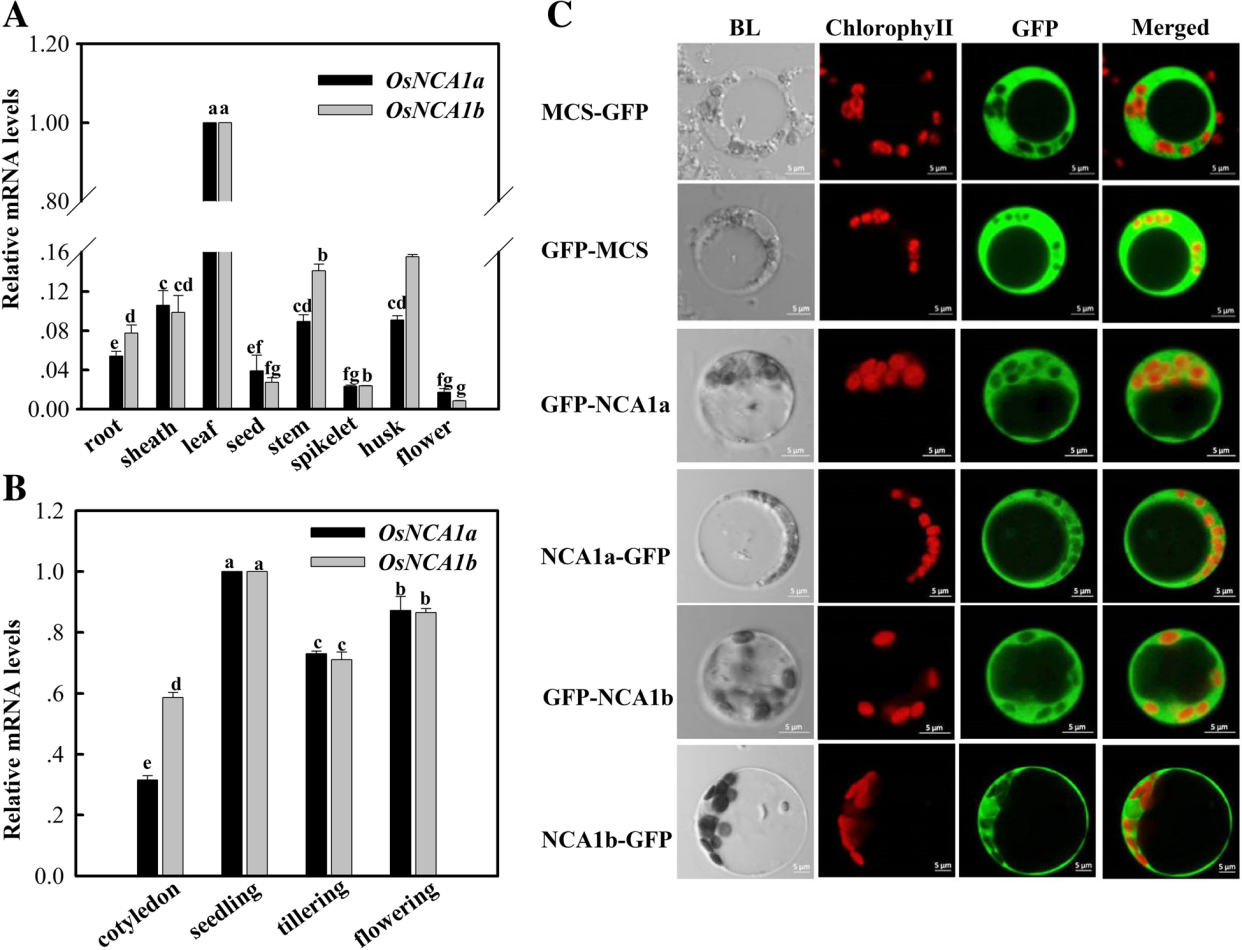
Spatiotemporal expression patterns of *OsNCA1a and OsNCA1b* and subcellular localization of OsNCA1a and OsNCA1b in rice. (A) Transcriptional expression in different organs. Different organs throughout the life cycle were sampled and analyzed by quantitative RT-PCR. Relative mRNA levels in different organs were graphed based on the *NCA1* mRNA level in leaf as 1. (B) Transcriptional expression in leaves at different growth stages. Cotyledons at budding stage and the second leaf from top at other stages were sampled for analysis. Relative mRNA levels at different growth stages were graphed based on the *NCA1* mRNA level in seeding stage as 1. Data are means (± SD) of three biological replicates. Different letters on the top of columns indicate significant difference at *p* < 0.05 according to Duncan’s multiple range test. (C) Subcellular localization as detected by protoplast transient expression analysis. OsNCA1a and OsNCA1b are located in the cytosol. Cells are imaged by a confocal microscope at 14–16 h after transfection; MCS, multiple cloning sites; MCS-GFP, GFP linked to C terminal; GFP-MCS, GFP linked to N terminal; NCA1a-GFP, GFP linked to C terminal of OsNCA1a; GFP-NCA1a, GFP linked to N terminal of OsNCA1a. Legends for OsNCA1b are the same as for OsNCA1a. BL, bright light; chlorophyll, chloroplast chlorophyll autofluorescence; GFP, GFP fluorescence. Scale bars, 5 μm. These results are representative of three independent experiments

*OsNCA1a* and *OsNCA1b* were distinctly expressed in different organs, with the highest abundance in leaves (Fig. 1a). Similar transcript levels were detected in rice leaves at different growth stages, except lower in the cotyledon (Fig. 1b). It was also noticed that the two genes displayed very similar expression patterns, pointing towards a possibility that *OsNCA1a* and *OsNCA1b* may have functioned redundantly. To examine the subcellular localization of the two proteins, both C-terminal and N-terminal GFP-tagged *OsNCA1a* and *OsNCA1b* were generated and transfected to rice protoplasts. Confocal microscopy observation revealed that both OsNCA1a and OsNCA1b are localized in the cytosol (Fig. 1c), consistent with the location of AtNCA1 in *Arabidopsis* [5].

### OsNCA1a and OsNCA1b can activate CAT equally

**Fig. 2:**
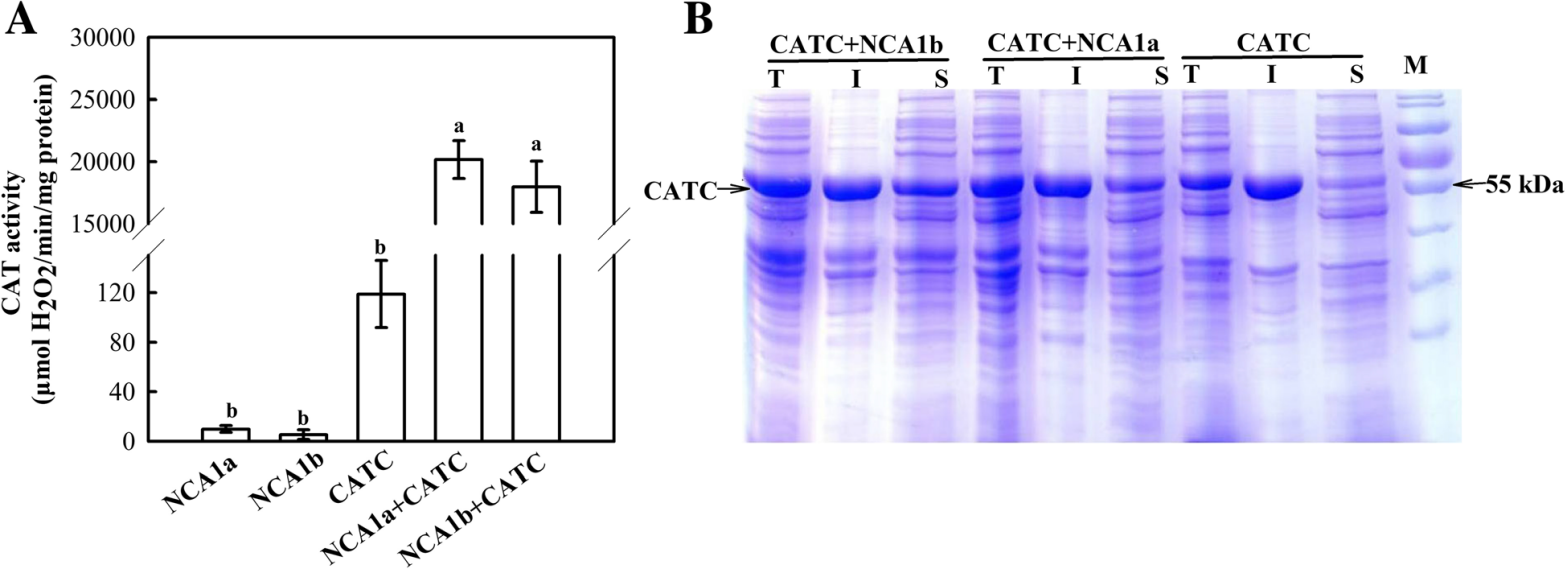
Effects of OsNCA1a and OsNCA1b on the activity and solubility of CATC as expressed in *E. coli*. (A) Effects of OsNCA1a and OsNCA1b on CAT activity. After proteins were inductively expressed, they were purified by passing a Ni-affinity column and then CAT activities were assayed; NCA1a, NCA1b, CATC: each was expressed alone; NCA1a + CATC or NCA1b + CATC: both were co-expressed; Data represent means ± SD of at least three replicate experiments. Different letters on the top of columns indicate significant difference at *p* < 0.05 according to Duncan’s multiple range test. (B) Proteins from supernatants, precipitates and whole *E. coli* cells were separately sampled and subjected to SDS-PAGE. I: insoluble (precipitate); S: soluble (supernatant); T: total proteins in whole cells. Molecular weight ladders (M) were loaded on the right lane

Only one *NCA1* gene exists in *Arabidopsis*, which was shown to regulate CAT activity by maintaining the folding of CAT [5], whereas there are two homologs in rice (Additional file 2 Figure S1). Since transcriptional analysis (Fig. 1) has implicated that both *NCA1*genes may have functioned similarly in rice, whether the two isoforms can activate CAT equally or differently remains a next interesting question. As shown in Fig. 2a, when *OsCATC* was expressed alone in *E. coli*, CAT activity was relatively low, with a specific activity of around 120 μmol H_2_O_2_ min^− 1^ mg^− 1^ protein; when either *OsNCA1a* or *OsNCA1b* was co-expressed with *OsCATC*, CAT activity was increased by more than 160 folds, up to 20,000 μmol H_2_O_2_ min^− 1^ mg^− 1^ protein. This result clearly indicates that either OsNCA1a or OsNCA1b is able to equally activate CAT in rice. It was meanwhile noticed that, when *OsCATC* was expressed alone, most of CAT proteins were contained in the inclusion body as insoluble proteins (Fig. 2b); when either *OsNCA1a* or *OsNCA1b* was co-expressed with *OsCATC*, much more CAT proteins were distributed in the supernatant as soluble proteins (Fig. 2b). This result means that both OsNCA1a and OsNCA1b can help CAT to fulfill a correct folding, based on the fact that proteins with correct folding will become more soluble and appear in the supernatant in *E. coli*, vice versa [23].

### OsNCA1a and OsNCA1b show mutually exclusive interaction with CAT

**Fig. 3:**
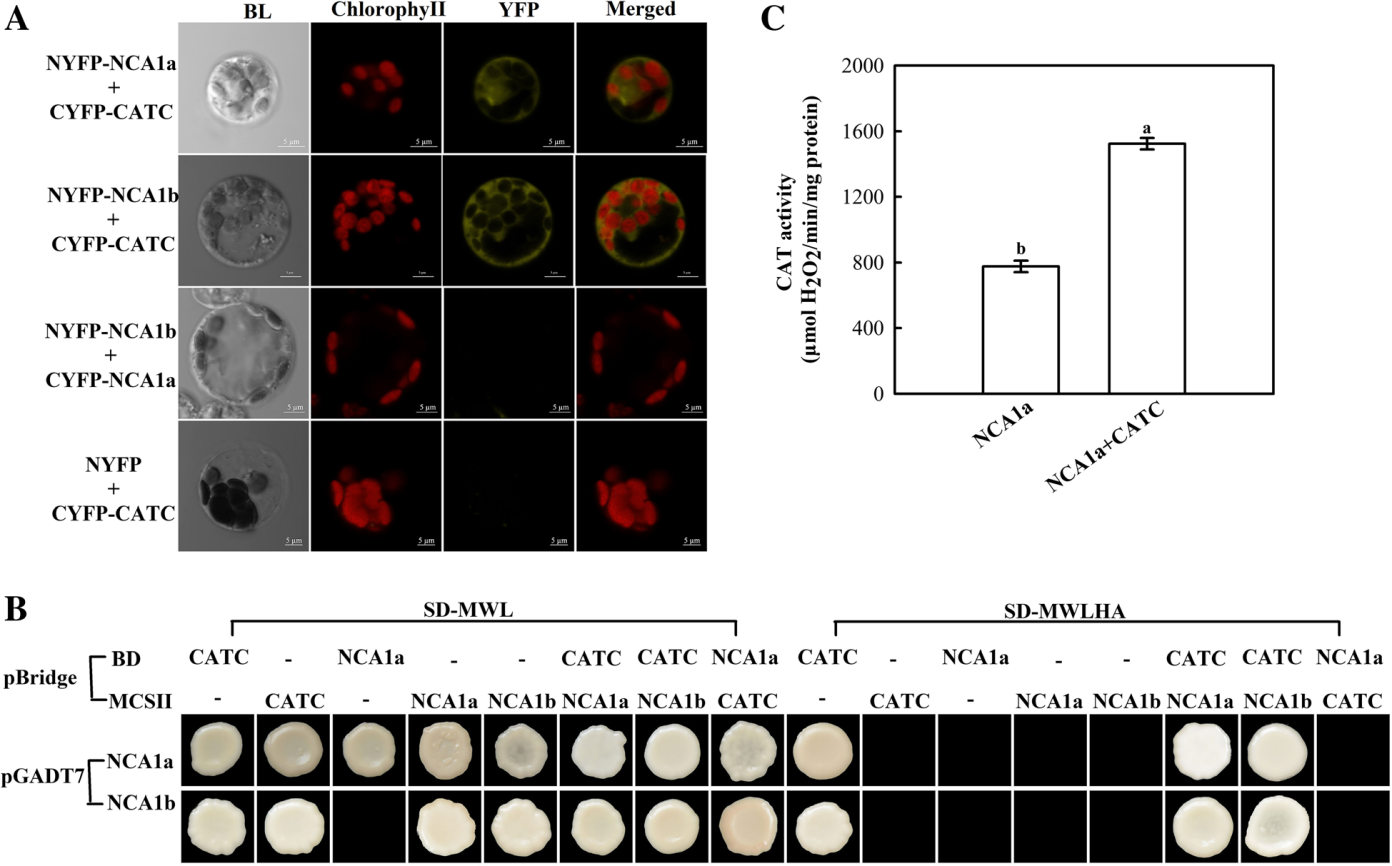
Interactions of CAT with OsNCA1a and / or OsNCA1b. (A) BiFC assay. NCA1 interacts with CATC in the cytosol. Plasmids were introduced into rice protoplasts and fluorescence was detected by confocal microscopy. Top row, *NYFP-NCA1a* and *CYFP-CATC* were cotransformed. Second row, *NYFP-NCA1b* and *CYFP-CATC* were cotransformed. Third row, *NYFP-NCA1b* and *CYFP-NCA1a* were cotransformed. Bottom row, *NYFP* vectors and *CYFP-CATC* were cotransformed. In each row, the first panel, bright-field image (BL); the second panel, chlorophyll autofluorescence (chlorophyll) in red; the third panel, YFP signal in yellow; and the fourth panel, merged image (Merge). Scale bars, 5 μm. (B) Yeast three-hybrid assay 4. Prey (pGADT7) and bait (pBridge) constructs were transformed into Y2Hgold yeast strain solely or together to examine the interactions. pBridge contains two distinct multiple cloning sites (MCS) allow expression of the BD (DNA binding domain) fusion as well as a third protein. “pBridge BD and pBridge MCSII” represent the first and the second MCS link gene in pBridge vector, and expressed separately in yeast. “-” represent pBridge BD or pBridge-MCSII without any gene fusion was used as a control. The pGADT7-NCA1a and pGADT7-NCA1b indicated that the pGADT7 AD (DNA Activation Domain) vector MCS were connected to the *NCA1a, NCA1b* respectively and expressed in yeast Y2Hgold cells. The picture shows yeast growth on selective dropout media lacking Methionine/Tryptophan/Leucine (SD-MWL) or Methionine/Tryptophan/Leucine/Histidine/Alanine (SD-MWLHA) to check for interactions between the three partners. (C) The activity of CATC from pBridge MCSII site. NCA1a: the yeast transformed with pBridge BD-NCA1a + MCSII-NCA1a and pGADT7-NCA1a grow on SD-MWL; NCA1a + CATC: the yeast transformed with pBridge BD-NCA1a + MCSII-CATCa and pGADT7-NCA1a grow on SD-MWL. The CAT activities of the total protein extracted from yeast cells were measured. Data represent means ± SD of at least three replicate experiments. Different letters on the top of columns indicate significant difference at *p* < 0.05 according to Duncan’s multiple range test

First, the BiFC experiment was conducted using rice protoplast transient expression system. As shown in Fig. 3a, both NCA1 isoforms can interact with CAT, while interaction did not occur between NCA1a and NCA1b. The next question is whether both isoforms interact simultaneously, or either interacts alternately, with CAT to realize the function. To answer this question, yeast three-hybrid assay was carried out. The results showed that both NCA1a and NCA1b could not interact with either NCA1a or NCA1b (the yeast transformed with pBridgeBD-NCA1a and pGADT7-NCA1a/pGADT7-NCA1b could not grow on SD-MWLHA media), suggesting that one single molecule of either NCA1a or NCA1b is the functional form to activate CAT (Fig. 3b). Furthermore, the results may also implicate that CAT has only one binding domain to interact with one molecule of NCA1 and is unable to act as a scaffold protein for more than one NCA1 molecule to drive the reporter gene expression in yeast three-hybrid system (the yeast transformed with pBridgeBD-CATC and pGADT7-NCA1a / pGADT7-NCA1b could grow on SD-MWLHA media, while the yeast transformed with pBridgeBD-NCA1a + MCSII-CATC and pGADT7-NCA1a / pGADT7-NCA1b could not grow on SD-MWLHA media; the expression of CATC from MCSII site was verified by CAT activity measurement) (Fig. 3b and c). Thus it can be further inferred that CAT interacts with either NCA1a or NCA1b in a molecular ratio of 1:1, in support of the previous result [5].

### Phenotypes and CAT activity of the *nca1a/nca1b* single and double mutants

**Fig. 4:**
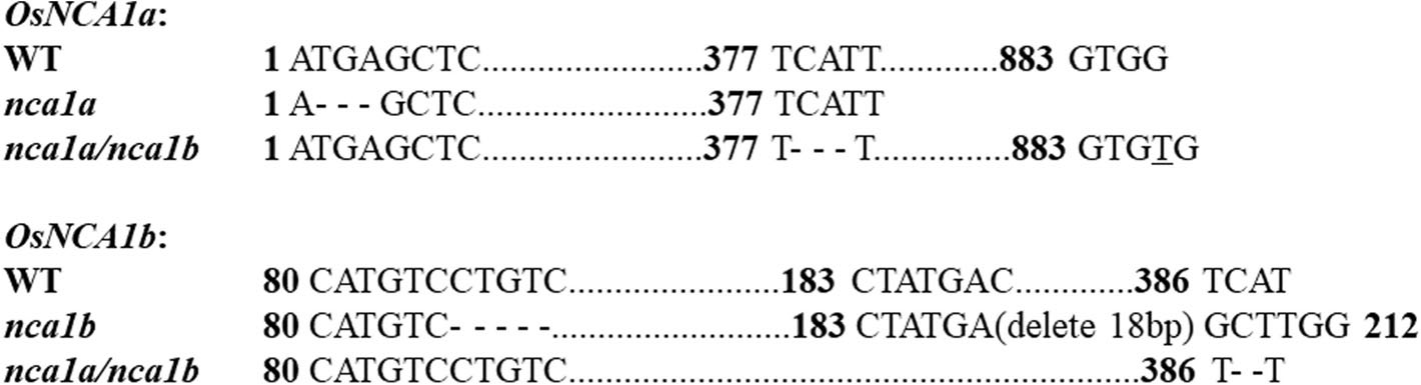
Molecular evaluation of the CRISPR-Cas9 generated *nca1a/nca1b* single and double mutants. The nucleotide underlined with “_” indicates mutations with the nucleotide newly inserted. “-” stands for deletion of nucleotides; Numbers with black character indicate the nucleotide position counting from ATG of the open reading frame

**Fig. 5:**
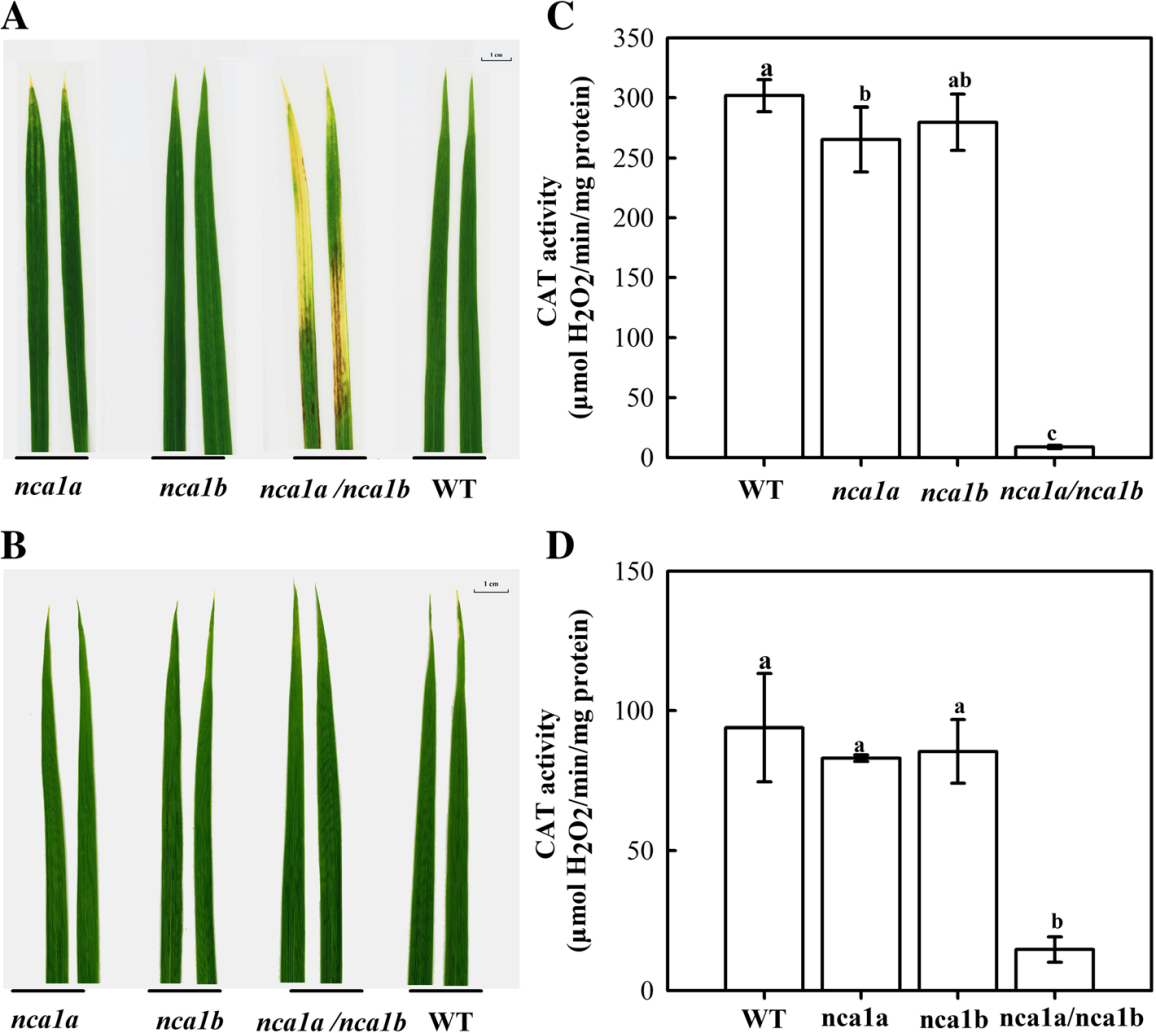
Phenotypes and CAT activity of the *nca1a/nca1b* single and double mutants under normal air and high CO_2_ conditions. The plants were hydroponically grown in an environment-controlled growth chamber for 15 d (temperature: 30 °C day/25 °C night; light intensity: 600 μmol m^− 2^ s^− 1^; humidity: 60%), under normal air condition 400 ppm CO_2_ (A); air enriched with 3500 ppm CO_2_ (B); CAT activity under normal air condition (C); CAT activity under 3500 ppm CO_2_ (D). Data represent means ± SD of at least three replicate experiments. Different letters on the top of columns indicate significant difference at p < 0.05 according to Duncan’s multiple range test

In order to further reveal the physiological function of *NCA1a* and *NCA1b* in rice, their single and double mutants were generated by CRISPR/Cas9. Sequence alignment of every sgRNA targeting region revealed that the three types of mutants were obtained, respectively, with *NCA1a* or *NCA1b* or both mutated (Fig. 4). Under normal conditions, either the *nca1a* or *nca1b* single mutant showed no any phenotypic difference throughout the growth period as compared with WT, whereas the *nca1a/nca1b* double mutant displayed obvious lesion and even albino symptoms on leaves throughout the growth stages (Fig. 5a). When the double mutant was grown under high CO_2_ (3500 ppm), which suppresses photorespiration and hence H_2_O_2_ production, the lesion phenotype was abolished (Fig. 5b), though CAT activity was still decreased by more than 95% in the double mutant, with no significant difference between air and high CO_2_ conditions (Fig. 5c and d). Therefore, the lesion phenotype of the *nca1a/nca1b* double mutant may have resulted from the accumulated photorespiratory H_2_O_2_.

### Effect of salinity stress on the *nca1a/nca1b* single and double mutants

**Fig. 6:**
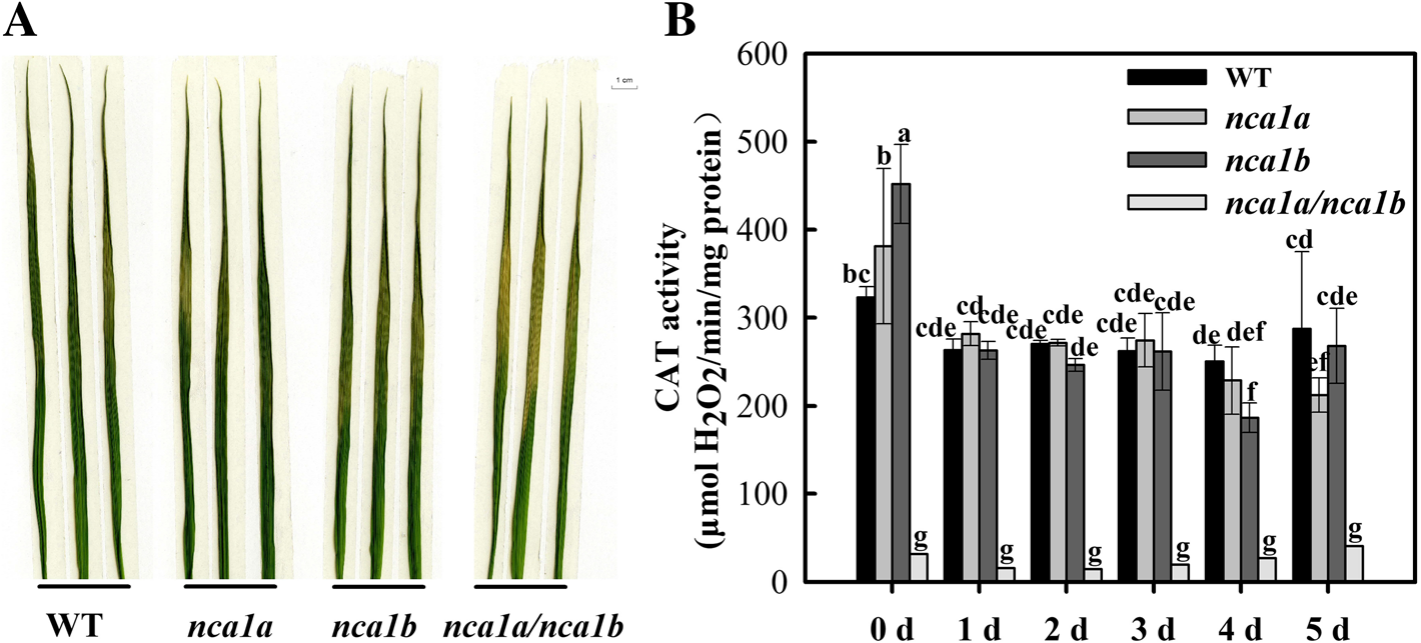
Phenotype and CAT activity for the *nca1* mutants in response to salinity stress. The plants were hydroponically grown in an environment-controlled growth chamber until 3.5-leaf old (temperature: 30 °C day/25 °C night; light intensity: 600 μmol m^− 2^ s^− 1^; humidity: 60%), then treated with 150 mM NaCl. CAT activity was assayed at different times after the treatment (B) and the picture of the stressed leaves was taken at 5 days after the treatment (A). Data represent means ± SD of at least three replicate experiments. Different letters on the top of columns indicate significant difference at p < 0.05 according to Duncan’s multiple range test

Only one *NCA1* gene exists in *Arabidopsis* and its mutation has been shown to be hypersensitive to salinity [5]. Here we tested effect of salinity stress on the *nca1a/nca1b* single and double knockout mutants of rice. First, phenotypic observation showed that no obvious difference occurred between either of the single mutants and WT under salinity stress, although the double mutant was shown to be more seriously damaged as compared with WT (Fig. 6a). Activity assay showed that the double mutant still had only 5% CAT activity of WT plants, similar to the result under normal condition (data not shown), whereas activity in either of the single mutants was not significantly different from WT plants (Fig.6b).

## Discussion

Catalase (CAT) is an important antioxidant enzyme in various organisms. Usually there exist 3 CAT genes in plant genomes, including *Arabidopsis*, rice, tobacco, maize, pumpkin etc. [1]. Besides scavenging H_2_O_2_, CAT plays essential roles in plant growth, development, and response to stress [1]. In *Arabidopsis*, knockout of *CAT2* leads to 80% loss of catalase activity and it is hypersensitive to salinity, strong light, and low temperature, but tolerance to LiCl and Hydroxyurea (HU) [5, 24, 25]. Rice CATC accounts for the large majority of catalase activity, and it is distributed mostly in rice leaves [9]. Since CAT protein are abundant in plants, overexpression of CAT genes normally does not significantly increase plant catalase activity or affect plant growth and response to stress. However, there have been a few studies showing that introduction of bacterial catalases into plants may increase plant catalase activity and plant resistance to oxidative stress, NaCl, and high light [1]. Despite the importance of this enzyme, its regulatory mechanism, particularly at the protein level, is not well understood. NCA1 protein was recently identified, which was shown to serve as a chaperone to activate CAT by helping it to fold correctly [5, 7]. The NCA1 gene exists in higher plants, with species having one gene and some harboring two copies (Additional file 2 Figure S1). According to the phylogenetic tree, there appears no regular pattern for its classification and evolution. Whether the two isoforms in those two-copy-containing plant species function redundantly or differently in regulating CAT activity and how they realize the function are not yet known. As a monocotyledonous model plant, rice harbors two *NCA1* genes, so it is particularly interesting to address the above question in rice. First of all, our spatiotemporal expression pattern analysis, in combination with biochemical studies, demonstrates that OsNCA1a and OsNCA1b could function equally in activating CAT (Fig. 1; Fig. 2).

Subsequent BiFC and yeast three-hybrid experiments established that OsNCA1a and OsNCA1b showed mutually exclusive interaction with CAT in cytosol (Fig. 3; Fig. 4). CAT is a peroxisomal matrix protein and the nascent peroxisomal proteins are translated on the cytoplasmic free polysomes [9], so the OsNCA1a and OsNCA1b could regulate the CAT activity in the cytosol, this is consistent with the results of Li et al. (2015) [5]. Furthermore, they also detected that the nascent CAT in the cytosol interacts with NCA1 and form complexes in a 1:1 ratio. Our yeast three-hybrid result still indicated that in rice CAT interacts with either NCA1a or NCA1b in a molecular ratio of 1:1. It is currently not clear how CAT interacts with NCA1. Previous studies have shown that the TPR domain functions as a protein interaction scaffold for the formation of different protein complexes [26].

Li et al. (2015) also reported that *Arabidopsis* defective of NCA1 was highly sensitive to salinity stress [5]. We also have originally imagined that, in those plant species containing two *NCA1* genes, the two *NCA1* genes may be simultaneously required under certain circumstances, likely under adverse conditions. However, by using *nca1a/nca1b* single and double knockout mutants, it was revealed that both genes function redundantly under both normal and salinity stress conditions (Fig. 5; Fig. 6). Such functionally-redundant genes have been frequently noticed in organisms. For instance, Blanvillain et al. (2008) identified two *XPO1 (EXPORTIN1)* genes in *Arabidopsis*, i. e, *XPO1a* and *XPO1b*, which were shown to be functionally redundant to regulate the maternal-to-embryonic transition [31]. Two TRAF (Tumor necrosis factor receptor-associated factor) proteins MUSE 13 and MUSE14 were identified in *Arabidopsis*, which serve as redundant immune regulators [32, 33]. It is not yet understood why such redundant genes/proteins exist naturally, but we believe that the two *NCA1* genes/proteins may be simultaneously required under certain circumstances yet to be identified, based on the previous reports that CAT activity can be regulated by various stresses including drought, cold, H_2_O_2_, methyl viologen (MV), high light or heat shock.

## Conclusions

Our study found that in rice there exist two NCA1 isoforms (NCA1a and NCA1b) that interact with CAT in a mutually exclusive manner to function-redundantly regulate CAT activity. It has been further demonstrated that CAT interacts with either NCA1a or NCA1b in a molecular ratio of 1:1. While redundant genes/proteins occur commonly in organisms, our study newly noticed that the two homologous proteins interact in a mutually exclusive manner to realize their function. We believe that the two NCA1 genes/proteins may be simultaneously required under certain circumstances yet to be identified, based on the previous reports that CAT activity can be regulated by various stresses including drought, cold, H_2_O_2_, MV, high light or heat shock [27–30].

## Methods

### Plant materials and growth conditions

Rice (*Oryza sativa* L) cv. Zhonghua 11 (japonica cultivar group) kept by our laboratory was used for generating the *NCA1a/NCA1b* single and double knockout mutants and for the functional analyses. Pre-germinated seeds were grown in Kimura B complete nutrient solution in a greenhouse condition [34]. Conditions of each treatment in growth chamber were specified in the legend of each figure.

### Plasmid construction

The complete ORFs of *OsNCA1a* (Os01g0104100) and *OsNCA1b* (Os02g0795300) were cloned from rice leaves by RT-PCR, the complete ORFs of Os*CATC* (Os03g0131200) were cloned from expression vectors described previously [9], and then the ORFs of *OsNCA1a*, *OsNCA1b*, and *OsCATC* were subcloned into the *pBridge* and *pGADT7* vectors for yeast three hybrid assay (Biosciences Clontech, Palo Alto, USA). For expression of the recombinant proteins in *E. coli*, the *OsNCA1a/b* sequences were inserted into the *pET28a* vector at the *Bam*H I and *Xho* I sites and *OsCATC* sequence was inserted into *pETDuet-1* first MCS between the *Eco*R I and *Hin*d III sites. For subcellular localization, the ORFs of *OsNCA1a*, *OsNCA1b* were introduced into *pYL322-DI*, under the control of the CAMV35S promoter. The *pYL322-DI* vector was kindly provided by Dr. Yao-Guang Liu, College of Life Sciences, South China Agricultural University. For constructing BiFC vectors, the ORFs of *OsNCA1a*, *OsNCA1b* and *OsCATC* were inserted into *pSAT6-cEYFP-C1* (CYFP) vector or *pSAT6-nEYFP-C1* (NYFP) vector [36]. The primers used for PCR were listed in Additional file 3 Table S2.

### Subcellular localization and bimolecular fluorescence complementation (BiFC)

Rice protoplasts were isolated as previously described Zhang et al. (2011) [35]. For subcellular localization, 10 μg of the *pYL322-DI*-*OsNCA1a/OsNCA1b*-GFP constructs was introduced into 100 μL protoplasts by the PEG-mediated transfection, and then the protoplasts were incubated in dark at 25 °C for 16–24 h.

For BiFC, vector of *pSAT6-cEYFP-C1* (CYFP); vector of *pSAT6-nEYFP-C1* (NYFP); *CYFP-CATC + NYFP-NCA1a/NCA1b*: *CATC* fused with *eYFP* at the C-terminal and *NCA1a/NCA1b* with *eYFP* at the N-terminal were then co-transfected into rice protoplasts; *CYFP-NCA1a + NYFP-NCA1b*: *NCA1a* fused with *eYFP* at the C-terminal and *NCA1b* with *eYFP* at the N-terminal were then transfected into rice protoplasts; *CYFP-CATC + NYFP*: *CATC* fused with *eYFP* at the C-terminal and *NYFP* as a blank vector were transfected into rice protoplasts. 5 μg of *NYFP*-tagged constructs and 5 μg *CYFP*-tagged constructs were co-transfected into 100 μL protoplasts by the PEG-mediated transfection. Then, the protoplasts were incubated in dark at 25 °C for 12 h [35, 36]. The confocal images were finally captured using the ZEISS LSCM 780 system.

### RNA isolation and qRT-PCR

Total RNA was extracted from rice leaves using Trizol reagent (Life Technologies, USA), and treated with RNase free-DNaseI (Amersham, USA). The quality and quantity of the purified RNA was assessed with a NanoDrop-1000 (NanoDrop, USA). First-strand cDNA was synthesized using ReverTra Ace (Toyobo, Japan). Specific primer pairs were designed for the qRT-PCR of each *NCA1* gene, and the specificity of these primers were evaluated using NCBI Primer-BLAST (Additional file 3 Table S2). The qRT-PCR was performed in 10 μL of reaction mixture consisting of 5 μL of 2 × SYBR Green PCR Master Mix (Toyobo, Japan), 0.2 μM of each primer, and 2 μL of appropriate diluted cDNA. Transcript levels of each gene were measured by the DNA Engine Opticon2 Real-Time PCR detection system and opticon monitor software (Bio-Rad, USA) according to the manufacturer’s instructions. The data were normalized to the amplification of the Os*Actin1* gene (Os03g0718100). Method of presenting quantitative real-time PCR data is the comparative C_T_ method (2^-ΔΔC^_T_ method).

### Expression of the recombinant proteins in *E. coli* and their purification

The *pET28a-OsNCA1a*, *pET28a-OsNCA1b*, *pETDuet1-NhisCATC*, and *pETDuet1-NhisCATC-OsNCA1* plasmids were transformed into *E. coli* Rosseta (DE3) cells and screened on Luria-Bertani (LB) plates containing kanamycin or ampicillin, and then colony PCR was performed. Protein expression was induced at 16 °C with 1 mM of isopropyl-thio-β-D-1-thiogalactopyranoside (IPTG) overnight. The recombinant protein was purified with nickel column according to the manufacturer’s protocol. The resulting proteins were separated using SDS-PAGE.

### Enzyme assays and protein measurement

The CAT activity was measured using a UV-spectrophotometer 20 in a reaction mixture containing 50 mM PBS (pH 7.4), 25 mM H_2_O_2_ at 30 °C. The consumption of H_2_O_2_ was detected at 240 nm and the CAT activity was calculated using the extinction coefficient for H_2_O_2_ of 43.6 M^− 1^ cm^− 1^ [37]. The protein contents were determined using Coomassie brilliant blue G250 [38].

### Generation *nca1/nca2* single and double knockout mutants by CRISPR/Cas9

The *nca1a/nca1b* single and double mutant was generated using CRISPR/Cas9 method according to Ma et al. (2015) [39]. First, two sgRNAs targeting each *OsNCA1* at different locations were cloned into *pYLsgRNA-OsU6a* and *pYLsgRNA-OsU6b*, respectively, then the vector *pYLCRISPR/Cas9 pubi-H* containing two U6-gRNA units for *OsNCA1a/ OsNCA1b* was constructed and transformed into rice via Agrobacteria tumefaciens (strain EHA105). Eight independent lines were obtained, respectively, to examine the function of CRISPR/Cas9. The genomic DNA was extracted from transgenic plants and primer pairs flanking the designed target site were used for PCR amplification. The amplicons were sequenced and sequence alignment of every sgRNA targeting region revealed that three independent mutants (*nca1a, nca1b, and nca1a/nca1b*) are reliable with loss of function of the single and double target genes (Fig. 4b).

### Yeast three hybrid assay

Yeast cultures and three-hybrid procedures were carried out according to standard methods [40]. In the yeast three-hybrid assay cells were transformed with the bicistronic vectors *pBridge* and *pGADT7* (Biosciences Clontech, Palo Alto, USA). *pBridge* contains two distinct multiple cloning sites to allow expression of the BD (DNA binding domain) fusion as well as a third protein. When pBridge is used in conjunction with the *pGADT7 AD* (DNA activation domain) fusion vector a ‘three-hybrid’ system can be established that is dependent on the expression of a third protein. Co-transformation of appropriate *BD* and *AD* constructs (*AD*-*NCA1a*, *AD*-*NCA1b*, *BD*-*CATC*, BD-*NCA1a*, *BD*-*NCA1b*) into the yeast Y2Hgold cells (Biosciences Clontech, Palo Alto, USA). Yeast transformed with both bait (BD) and prey (AD) vectors were spotted on selective dropout media without Methionine/Tryptophan/Leucine (SD-MWL) or Methionine/Tryptophan/Leucine/ Histidine/Alanine (SD-MWLHA) to check for interaction between the three partners. In each case, a control with an empty vector was tested for self-activation of the constructs.

### Statistical analysis

The data were subjected to statistical analysis using Duncan’s multiple range test at the 5% (*P* < 0.05) confidence levels. Data Processing System (DPS) software [41].were used for data statistics analysis.

## Additional files

Additional file 1: **Table S1.** Similarities of *Arabidopsis* and rice GLO genes at the level of mRNA and protein. (DOCX 58 kb)

Additional file 2: **Figure S1.** Phylogenetic relationships among NCA and orthologous proteins from other species. (DOCX 18 kb)

Additional file 3: **Table S2.** Primers used in this study. (DOCX 20 kb)

## Abbreviations

BiFC: Bimolecular fluorescence complementation
CAT: Catalase
IPTG: Isopropyl-thio-β-D-1-thiogalactopyranoside
NCA1: NO CATALASE ACTIVITY 1
qRT-PCR: Real-time quantitative PCR

## References

[1] Mhamdi A, Noctor G, Baker A (2012). Plant catalases: peroxisomal redox guardians. Arch Biochem Biophys.

[2] Regelsberger G, Jakopitsch C, Plasser L, Schwaiger H, Furtmuller PG, Peschek GA, Zamocky M, Obinger C (2002). Occurrence and biochemistry of hydroperoxidases in oxygenic phototrophic prokaryotes (*cyanobacteria*). Plant Physiol Biochem.

[3] Iwamoto M, Higo H, Higo K (2000). Differential diurnal expression of rice catalase genes: the 5′-flanking region of CatA is not sufficient for circadian control. Plant Sci.

[4] Mhamdi A, Queval G, Chaouch S, Vanderauwera S, Van Breusegem F, Noctor G (2010). Catalase function in plants: a focus on *Arabidopsis* mutants as stress-mimic models. J Exp Bot.

[5] Li J, Liu JT, Wang GQ, Cha JY, Li GN, Chen S, Li Z, Guo JH, Zhang CG, Yang YQ (2015). A chaperone function of no catalase activity1 is required to maintain catalase activity and for multiple stress responses in *Arabidopsis*. Plant Cell.

[6] Chaouch S, Noctor G (2010). Myo-inositol abolishes salicylic acid-dependent cell death and pathogen defence responses triggered by peroxisomal hydrogen peroxide. New Phytol.

[7] Hackenberg T, Juul T, Auzina A, Gwizdz S, Malolepszy A, Van Der Kelen K, Dam S, Bressendorff S, Lorentzen A, Roepstorff P (2013). Catalase and no catalase activity1 promote autophagy-dependent cell death in *Arabidopsis*. Plant Cell.

[8] Vanderauwera S, Zimmermann P, Rombauts S, Vandenabeele S, Langebartels C, Gruissem W, Inze D, Van Breusegem F (2005). Genome-wide analysis of hydrogen peroxide-regulated gene expression in *Arabidopsis* reveals a high light-induced transcriptional cluster involved in anthocyanin biosynthesis. Plant Physiol.

[9] Zhang Z, Xu Y, Xie Z, Li X, He ZH, Peng XX (2016). Association-dissociation of glycolate oxidase with catalase in rice: a potential switch to modulate intracellular H_2_O_2_ levels. Mol Plant.

[10] Lin A, Wang Y, Tang J, Xue P, Li C, Liu L, Hu B, Yang F, Loake GJ, Chu C (2012). Nitric oxide and protein S-nitrosylation are integral to hydrogen peroxide-induced leaf cell death in rice. Plant Physiol.

[11] Redinbaugh MG, Sabre M, Scandalios JG (1990). Expression of the maize Cat3 catalase gene is under the influence of a circadian rhythm. P Natl Acad Sci USA.

[12] Queval G, Issakidis-Bourguet E, Hoeberichts FA, Vandorpe M, Gakiere B, Vanacker H, Miginiac-Maslow M, Van Breusegem F, Noctor G (2007). Conditional oxidative stress responses in the *Arabidopsis* photorespiratory mutant *cat2* demonstrate that redox state is a key modulator of daylength-dependent gene expression, and define photoperiod as a crucial factor in the regulation of H_2_O_2_-induced cell death. Plant J.

[13] Xing Y, Jia WS, Zhangl JH (2008). AtMKK1 mediates ABA-induced CAT1 expression and H_2_O_2_ production via AtMPK6-coupled signaling in *Arabidopsis*. Plant J.

[14] Zimmermann P, Heinlein C, Orendi G, Zentgraf U (2006). Senescence-specific regulation of catalases in *Arabidopsis thaliana* (L.) Heynh. Plant Cell Environ.

[15] Dietrich RA, Richberg MH, Schmidt R, Dean C, Dangl JL (1997). A novel zinc finger protein is encoded by the *Arabidopsis* LSD1 gene and functions as a negative regulator of plant cell death. Cell.

[16] Li YS, Chen LC, Mu JY, Zuo JR (2013). Lesion simulating disease1 interacts with catalases to regulate hypersensitive cell death in *Arabidopsis*. Plant Physiol.

[17] Li G, Li J, Hao R, Guo Y (2017). Activation of catalase activity by a peroxisome-localized small heat shock protein Hsp17.6CII. J Genet Genomics.

[18] Zou JJ, Li XD, Ratnasekera D, Wang C, Liu WX, Song LF, Zhang WZ, Wu WH (2015). *Arabidopsis* calcium-dependent protein kinase8 and catalase3 function in abscisic acid-mediated signaling and H_2_O_2_ homeostasis in stomatal guard cells under drought stress. Plant Cell.

[19] Yang T, Poovaiah BW (2002). Hydrogen peroxide homeostasis: activation of plant catalase by calcium/calmodulin. P Natl Acad Sci USA.

[20] Fukamatsu Y, Yabe N, Hasunuma K (2003). *Arabidopsis* NDK1 is a component of ROS signaling by interacting with three catalases. Plant Cell Physiol.

[21] Verslues PE, Batelli G, Grillo S, Agius F, Mm YS, Zhu J, Agarwal M, Katiyar-Agarwal S, Zhu JK (2007). Interaction of SOS2 with nucleoside diphosphate kinase 2 and catalases reveals a point of connection between salt stress and H_2_O_2_ signaling in *Arabidopsis thaliana*. Mol Cell Biol.

[22] Mathioudakis MM, Veiga RS, Canto T, Medina V, Mossialos D, Makris AM, Livieratos I (2013). Pepino mosaic virus triple gene block protein 1 (TGBp1) interacts with and increases tomato catalase 1 activity to enhance virus accumulation. Mol Plant Pathol.

[23] Hartl FU (1996). Molecular chaperones in cellular protein folding. Nature.

[24] Bueso E, Alejandro S, Carbonell P, Perezamador MA, Fayos J, Bellés JM, Rodriguez PL, Serrano R (2007). The lithium tolerance of the *Arabidopsis* cat2 mutant reveals a cross-talk between oxidative stress and ethylene. Plant J.

[25] Juul T, Malolepszy A, Dybkaer K, Kidmose R, Rasmussen JT, Andersen GR, Johnsen HE, Jorgensen JE, Andersen SU (2010). The in vivo toxicity of hydroxyurea depends on its direct target catalase. J Biol Chem.

[26] Allan RK, Ratajczak T (2011). Versatile TPR domains accommodate different modes of target protein recognition and function. Cell Stress Chaperones.

[27] Gossett DR, Banks SW, Millhollon EP, Lucas MC (1996). Antioxidant response to NaCl stress in a control and an NaCl-tolerant cotton cell line grown in the presence of paraquat, buthionine sulfoximine, and exogenous glutathione. Plant Physiol.

[28] Dat JF, Lopez-Delgado H, Foyer CH, Scott IM (1998). Parallel changes in H_2_O_2_ and catalase during thermotolerance induced by salicylic acid or heat acclimation in mustard seedlings. Plant Physiol.

[29] Du YY, Wang PC, Chen J, Song CP (2008). Comprehensive functional analysis of the catalase gene family in *Arabidopsis thaliana*. J Integr Plant Biol.

[30] Distelbarth H, Nägele T, Heyer AG (2013). Responses of antioxidant enzymes to cold and high light are not correlated to freezing tolerance in natural accessions of *Arabidopsis thaliana*. Plant Biol.

[31] Blanvillain R, Boavida LC, Mccormick S, Ow DW (2008). Exportin1 genes are essential for development and function of the gametophytes in *Arabidopsis thaliana*. Genetics.

[32] Hung IH, Schoenwolf GC, Lewandoski M, Ornitz DM (2016). A combined series of Fgf9 and Fgf18 mutant alleles identifies unique and redundant roles in skeletal development. Dev Biol.

[33] Qi H, Xia FN, Xie LJ, Yu LJ, Chen QF, Zhuang XH, Wang Q, Li F, Jiang L, Xie Q (2017). TRAF family proteins regulate autophagy dynamics by modulating autophagy protein6 stability in *Arabidopsis*. Plant Cell.

[34] Yoshida S, Forno DA, Cock JH, Gomez KA. Laboratory manual for physiological studies of rice Manila: International Rice Research Institute; 1976.

[35] Zhang Y, Su J, Duan S, Ao Y, Dai J, Liu J, Wang P, Li Y, Liu B, Feng D (2011). A highly efficient rice green tissue protoplast system for transient gene expression and studying light/chloroplast-related processes. Plant Methods.

[36] Yoo SD, Cho YH, Sheen J (2007). *Arabidopsis* mesophyll protoplasts: a versatile cell system for transient gene expression analysis. Nat Protoc.

[37] Jiang ZY, Woollard AC, Wolff SP (1990). Hydrogen peroxide production during experimental protein glycation. FEBS Lett.

[38] Bradford MM (1976). A rapid and sensitive method for the quantitation of microgram quantities of protein utilizing the principle of protein-dye binding. Anal Biochem.

[39] Ma XL, Zhang QY, Zhu QL, Liu W, Chen Y, Qiu R, Wang B, Yang ZF, Li HY, Lin YR (2015). A robust CRISPR/Cas9 system for convenient, high-efficiency multiplex genome editing in monocot and dicot plants. Mol Plant.

[40] Gietz D, Jean AS, Woods RA, Schiestl RH (1992). Improved method for high efficiency transformation of intact yeast cells. Nucleic Acids Res.

[41] Tang Q, Zhang C (2013). Data processing system (DPS) software with experimental design, statistical analysis and data mining developed for use in entomological research. Insect Sci.

