# Supplementary material for: Two NCA1 isoforms interact with catalase in a mutually exclusive manner to redundantly regulate its activity in rice
Source: BMC Plant Biol. 2019 Mar 18;19:105. doi: 10.1186/s12870-019-1707-0 (PMC6421683; doi:10.1186/s12870-019-1707-0)
Supplement: Supplementary file 1 — Table S1. Similarities of Arabidopsis and rice GLO genes at the level of mRNA and protein. (DOCX 58 kb) [file 12870_2019_1707_MOESM1_ESM.docx]

**Fig.S1.**

***Oryza sativa 1 (*OsNCA1a*)***

***Oryza sativa 2* (OsNCA1b)**

***Oryzabrachyantha***

***Setariaitalica***

***Zea mays***

***Sorghum bicolor***

***Brachypodiumdistachyon 1***

***Brachypodiumdistachyon 2***

***Musa acuminata***

***Arabidopsis thaliana***

***Arabidopsis lyrata***

***Glycine soja 1***

***Glycine soja 2***

***Solanumlycopersicum***

***Solanumtuberosum***

***Nicotianatabacum 1***

***Nicotianatabacum 2***

***Physcomitrella patens***

100

100

99

99

99

24

52

71

99

99

93

50

98

84

97

0.050

| **Species** | **Accession number** | |
| --- | --- | --- |
| ***Oryza sativa 1*** | **XP_015617573.1** | **LOC4326292** |
| ***Oryza sativa 2*** | **XP_015627027.1** | **LOC4331003** |
| ***Oryza brachyantha*** | **XP_006643640.1** | **LOC102704921** |
| ***Setaria italica*** | **XP_004967927** | **LOC101770029** |
| ***Zea mays*** | **NP_001140913.1** | **LOC100272990** |
| ***Sorghum bicolor*** | **XP_002444897.1** | **LOC8064367** |
| ***Brachypodium distachyon 1*** | **XP_003559441.1** | **LOC100832811** |
| ***Brachypodium distachyon 2*** | **XP_003563481.1** | **LOC100836228** |
| ***Musa acuminata*** | **XP_009420238.1** | **LOC104000028** |
| ***Arabidopsis thaliana*** | **NP_191004.1** | **AT3G54360** |
| ***Arabidopsis lyrata*** | **XP_020880993.1** | **LOC9314041** |
| ***Glycine soja 1*** | **KHN26422.1** | **Glysoja_030376** |
| ***Glycine soja 2*** | **KHN45782.1** | **Glysoja_046420** |
| ***Solanum lycopersicum*** | **NP_001311399.1** | **LOC101263558** |
| ***Solanum tuberosum*** | **XP_006351347.1** | **LOC102597556** |
| ***Nicotiana tabacum 1*** | **XP_016464833.1** | **LOC107787735** |
| ***Nicotiana tabacum 2*** | **XP_016464837.1** | **LOC107787735** |
| ***Physcomitrella patens*** | **XP_024374425.1** | **LOC112281781** |

**Fig.S1. Phylogenetic relationships among NCA and orthologous proteins from other species.**

BLASTP was performed using the NCA1a protein sequence as query against NCBI nonredundant protein database. Orthologous proteins were identified from different organisms with a default cutoff E value. Highly homologous sequences selected from the representative species were aligned. In this tree, proteins are shown as the names of plant species followed by the corresponding accession numbers registered in the NCBI protein database. The evolutionary history was inferred by using the Maximum Likelihood method based on the Poisson correction model (Zuckerkandl and Pauling, 1965). Evolutionary analyses were conducted in MEGA7 (Kumar *et al.*, 2016).

Reference:

**Kumar S, Stecher G, Tamura K**. 2016. MEGA7: Molecular Evolutionary Genetics Analysis version 7.0 for bigger datasets. *Molecular Biology & Evolution* **33**, 1870.

**Zuckerkandl E, Pauling L**. 1965. Evolutionary Divergence and Convergence in Proteins. *Evolving Genes & Proteins* **97**, 97-166.
