# Supplementary material for: Two NCA1 isoforms interact with catalase in a mutually exclusive manner to redundantly regulate its activity in rice
Source: BMC Plant Biol. 2019 Mar 18;19:105. doi: 10.1186/s12870-019-1707-0 (PMC6421683; doi:10.1186/s12870-019-1707-0)
Supplement: Supplementary file 2 — Figure S1. Phylogenetic relationships among NCA and orthologous proteins from other species. (DOCX 18 kb) [file 12870_2019_1707_MOESM2_ESM.docx]

**Table S1. Similarities of *arabidopsis* and rice GLO genes at the level of mRNA and protein.**

| **mRNA**  **Prorein** | **AtNCA1** | **OsNCA1a** | **OsNCA1b** |
| --- | --- | --- | --- |
| **AtNCA1** |  | **63.9%** | **57.3%** |
| **OsNCA1a** | **59.8%** |  | **94.5%** |
| **OsNCA1b** | **58.4%** | **92.7%** |  |
