# Supplementary material for: Two NCA1 isoforms interact with catalase in a mutually exclusive manner to redundantly regulate its activity in rice
Source: BMC Plant Biol. 2019 Mar 18;19:105. doi: 10.1186/s12870-019-1707-0 (PMC6421683; doi:10.1186/s12870-019-1707-0)
Supplement: Supplementary file 3 — Table S2. Primers used in this study. (DOCX 20 kb) [file 12870_2019_1707_MOESM3_ESM.docx]

**Table S2. Primers used in this study.**

| Primer name | Primer sequence （5'→3'） | Purpose | |
| --- | --- | --- | --- |
| NCA1aS1A-U6a-F | GCCGGGGCATAGAGAGCTCATAA | CRISPR/Cas9 vector construction | |
| NCA1aS1A-U6a-R | AAACTTATGAGCTCTCTATGCCC |  |  |
| NCA1aS2A-U6b-F | GTTGTCATAGCCAAAGGGGCACTT |  |  |
| NCA1aS2A-U6b-R | AAACAAGTGCCCCTTTGGCTATGA |  |  |
| NCA1bS1-U6a-F | GCCGTTGGCTGTGACAGGACATG |  |  |
| NCA1bS1-U6a-R | AAACCATGTCCTGTCACAGCCAA |  |  |
| NCA1bS2-U6b-F | GTTGCCAAGCTTGAAGGTGTTGT |  |  |
| NCA1bS2-U6b-R | AAACACAACACCTTCAAGCTTGG |  |  |
| NCA1a/NCA1b-U6a-F | GCCGGAGCTGTGTTAGGAATGCT |  |  |
| NCA1a/NCA1b-U6a-R | AAACAGCATTCCTAACACAGCTC |  |  |
| NCA1a/NCA1b-U6b-F2 | GTTGGGCATGGCCATCAATGAAG |  |  |
| NCA1a/NCA1b-U6b-R2 | AAACCTTCATTGATGGCCATGCC |  |  |
| U-F | CTCCGTTTTACCTGTGGAATCG |  |  |
| gR-R | CGGAGGAAAATTCCATCCAC |  |  |
| HPT-F | CTGAACTCACCGCGACGTCTGTC | HPT gene amplification | |
| HPT-R | TAGCGCGTCTGCTGCTCCATACA |  |  |
| NCA1a-SZAD-*Bam*HI-F | GACTGGATCCATATGAGCTCTCTATGCCCCTTTGC | Yeast three-hybrid | |
| NCA1a-SZAD-*Xho*l-R | CATACTCGAGCTCACTTGTCATCCAATTGTTTTTG |  |  |
| NCA1b-SZAD-*Bam*HⅠ-F | GACTGGATCCATATGAGCTCTCTATGCCCGTTTGC |  |  |
| NCA1b-SZAD-*Xho*l-R | CATACTCGAGCTCACTTGTCATCCAGTTGTTTTTGT |  |  |
| CATC-SZ-*Bgl*Ⅱ-R | CATAAGATCTTTACATGCTCGGCTTCGCGCTG |  |  |
| CATC-Bridge2-Nhis-*Not*Ⅰ-F3 | GACTGCGGCCGCAATGCATCATCACCATCACCATATGG |  |  |
| CATC-Bridge1-CZ*Eco*RⅠ-F | TTGACTGTATCGCCGGAATTCATGGATCCCTACAAGCACCG |  |  |
| NCA1a-bridge1-CZ*Eco*RI-F | TTGACTGTATCGCCGGAATTCATGAGCTCTCTATGCCCCTT |  | |
| NCA1a-Bridge1-CZ*Eco*RI-R | TCGACGGATCCCCGGGAATTCTCACTTGTCATCCAATTGTT |  |  |
| NCA1b-pBridge2-nhis-cz*Not*I-F2 | AAGAAGAGAAAGGTGGCGGCCGCACATCATCACCATCACCATATG |  |  |
| NCA1b-pBridge2-CZ*Bgl*II-R | GGGAGATCAGCCCGAAGATCTTCACTTGTCATCCAGTTGTTTT |  |  |
| CATC-Bridge1-CZ*Pst*I-R | CCGGAATTAGCTTGGCTGCAGTTACATGCTCGGCTTCGCGC |  | |
| NCA1a-pBridge2-nhis-cz*Not*I-F | AAGAAGAGAAAGGTGGCGGCCGCACATCATCACCATCACCATATGAGCTCTCTATGCCCCTT |  | |
| NCA1a-pBridge2-CZ*Bgl*II-R | GGGAGATCAGCCCGAAGATCTTCACTTGTCATCCAATTGTT |  | |
| NCA1a_209-1280_cas9-2-F | CCATGCGATTTAATTCTATTCCCTA | Knockout lines identification | |
| NCA1a_209-1280_cas9-2-R | TGACTTTGCACTTTCAATGTTCTGT |  |  |
| NCA1b_4131-4986_cas9-2-F | CCGATTGGATTGTCTCATGTGCTGG |  |  |
| NCA1b_4131-4986_cas9-2-R | TGGTCTTGTCACTTGCGGCTTCACC |  |  |
| NCA1a/NCA1b_770-1607_cas9-F3 | CCCGTGCTCCCACAAGTT |  |  |
| ND1_770-1607_cas9-R3 | ATGATGCCATGCTATTAAAG |  |  |
| NCA1a/NCA1b_4378-5593_cas9-F3 | ACCAATCTTTTATGCAGG |  |  |
| NCA1a/NCA1b_4378-5593_cas9-R3 | TTTGACCACTCGTCTTTT |  |  |
| NCA1a-NGFP-*Kpn*I-F | GACTGGTACCATGAGCTCTCTATGCCCCTTTGC | NCA1 subcellular localization | |
| NCA1a-NGFP-*Sma*I-R | CATACCCGGGTCACTTGTCATCCAATTGTTTT |  |  |
| NCA1a-CGFP-*Kpn*I-F | GACTGGTACCGCATGAGCTCTCTATGCCCCTTTGC |  |  |
| NCA1a-CGFP-*Sma*I–R | CATACCCGGGCTTGTCATCCAATTGTTTTTGT |  |  |
| NCA1b-NGFP-*Kpn*I-F | GACTGGTACCATGAGCTCTCTATGCCCGTTTGC |  |  |
| NCA1b-NGFP-*Sma*I-R | CATACCCGGGTCACTTGTCATCCAGTTGTTTTTGT |  |  |
| NCA1b-CGFP-*Kpn*I-F | GACTGGTACCGCATGAGCTCTCTATGCCCGTTTGC |  |  |
| NCA1b-CGFP-*Sma*I-R | CATA CCCGGGCTTGTCATCCAGTTGTTTTTGTA |  |  |
| NCA1a-PET28-*Xho*I-R | CATACTCGAGTCACTTGTCATCCAATTGTTTTTG | | Prokaryotic expression |
| NCA1b-PET28-*Bam*HI-F | GACTGGATCCATGAGCTCTCTATGCCCGTTTG | |  |
| NCA1b-PET28-*Xho*I-R | CATACTCGAGTCACTTGTCATCCAGTTGTTTTTG | |  |
| NCA1a-NYFP-*Xho*I-F | CATACTCGAGCTATGAGCTCTCTATGCCCCTTTG | | NCA1 and CATC BIFC |
| NCA1a-NYFP-*Kpn*I-R | GACTGGTACCTCACTTGTCATCCAATTGTTTTTG | |  |
| NCA1b-NYFP-*Xho*I-F | CATACTCGAGCTATGAGCTCTCTATGCCCGTTTG | |  |
| NCA1b-NYFP-*Kpn*I-R | GACTGGTACCTCACTTGTCATCCAGTTGTTTTTG | |  |
| CATC-duet1-*Eco*RI-F | CATAGAATTCGATGGATCCCTACAAGCACCGC | | Prokaryotic expression |
| CATC-duet1-*Hin*dIII-R | GACTAAGCTTTTACATGCTCGGCTTCGCGCTG | |  |
| NCA1a-duet2-*Nde*I-F | GACTCATATGATGAGCTCTCTATGCCCCTTTGC | |  |
| NCA1a-duet2-*Xho*I-R | CATACTCGAGTCACTTGTCATCCAATTGTTTTTG | |  |
| NCA1b-duet2-*Nde*I-F | GACTCATATGATGAGCTCTCTATGCCCGTTTG | |  |
| NCA1b-duet2-*Xho*I-R | CATACTCGAG TCACTTGTCATCCAGTTGTTTTTG | |  |
| Actin-F | GGTATTGTTAGCAACTGGGATG | | qRT-PCR |
| Actin-R | GATGAAAGAGGGCTGGAAGA | |  |
| OSCATC-F | TCAAGAGATGGATCGACGCACTCTC | |  |
| OSCATC-R | GAAGCAGATTGCAACGCTGATCG | |  |
| OSNCA1a-F | CCTTCCTTCTCTTCTCCTCCT | |  |
| OSNCA1a-R | CGGTGTGATTGATGGTGGT | |  |
| OSNCA1b-F | GCTCTCTATGCCCGTTTGCCAA | |  |
| OSNCA1b-R | TCGGTGTGATTGTTGGCTGTGAC | |  |
